# Strangled gravidic uterus, an exceptional complication of umbilical hernia during pregnancy, a case report

**DOI:** 10.1016/j.amsu.2021.103143

**Published:** 2021-12-02

**Authors:** Rachid Jabi, Siham Elmir, Karam Saoud, Houda Mir Ali, Siham Nasri, Imane Skiker, Hanane Saadi, Brahim Housni, Mohammed Bouziane

**Affiliations:** a Department of General Surgery, Mohammed VI University Hospital, Faculty of Medicine and Pharmacy, Oujda, Morocco; b Laboratory of Anatomy, Microsurgery and Surgery Experimental and Medical Simulation LAMCESM, Mohammed Ist University, Oujda, Morocco; c Department of Physical Medicine and Rehabilitation, Mohammed VI University Hospital, Faculty of Medicine and Pharmacy, Oujda, Morocco; d Department of Gynecology and Obstetrics, Faculty of Medicine and Pharmacy Fes, Morocco; e Department of Radiology, Mohammed VI University Hospital, Faculty of Medicine and Pharmacy, Oujda, Morocco; f Department of Gynecology and Obstetrics, Mohammed VI University Hospital, Faculty of Medicine and Pharmacy, Oujda, Morocco; g Department of Anaesthesia and Intensive Care, Mohammed VI University Hospital, Faculty of Medicine and Pharmacy, Oujda, Morocco

**Keywords:** Umbilical hernia, SCARE, Strangled pregnancy, Sugery, Recurrence

## Abstract

**Introduction:**

Strangulated pregnancy is a very rare presentation in which the intra umbilical strangulated form is exceptional. To our knowledge, we report the first Moroccan case and one of less than 10 cases published in the literature of a strangulated gravid uterus; in a woman admitted for treatment of umbilical pain.

**Case presentation:**

Through this presentation, we report a sporadic case of hernial strangulation during pregnancy containing an evolving pregnancy in the umbilical harness bag. The suspicion of this diagnosis was clinical and the confirmation made by ultrasound and abdominal MRI for confirmation. The objectives of this publication are threefold: i), to report this new exceptional case ii), to highlight the place of imaging in the management of hernial pathology iii), and to recommend surgical treatment of umbilical hernias in women of childbearing age in order to avoid surgical complications and maternal and fetal morbidity and mortality.

**Conclusion:**

Our case report shows that we should consider this very rare presentation of strangulated pregnancy. Our work also reports another new case to the poor published literature on this subject and emphasizes the importance of surgical management of parietal pathology by focusing on the parietal impact of physiological change during pregnancy.

## Highlights

• Atypical presentation of the strangled uterus.
• A strangled umbilical hernia containing a pregnant uterus being one of less than 10 cases reported in the literature.
• Take into consideration the possibility of complication of parietal pathology during pregnancy.

## 1 Introduction

Visceral emergencies during pregnancy are a frequent reason for consultation [1]. While the clinical particularity of a pregnant woman considers the physiological changes and the transformation of the anatomical reference points [2], the management of any affections must call upon eliminating the obstetrical origin in first place [3].

In this work, we report according to SCARE guidelines a case of a 34-year-old woman admitted with abdominal pain. The clinical and radiological examinations showed umbilical strangulation of the gravid uterus which was be treated by placement of an intra-abdominal plate.

The particularity of our exceptional case is twofold: i) it is a rare case [4] and only 10 similar cases were published in the literature, ii) there is no standard procedure for such pathology.

We add another case to the poor literature published in this field which enriches the research and can probably direct the standardization of the management of its rare presentations.

## 2 Clinical case

**Fig. 1:**
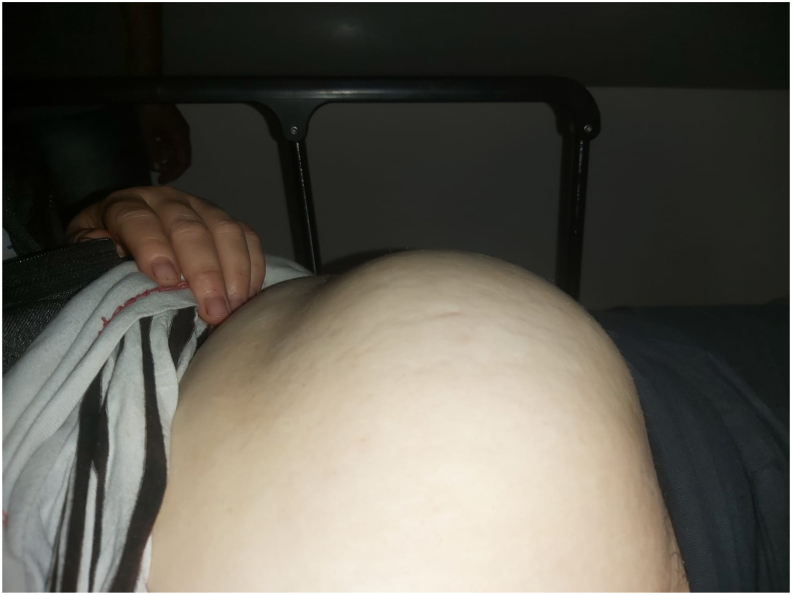
F1 clinical examination revealed a conscious patient with an irreducible and impulsive painful mass at the umbilical level.

**Fig. 2:**
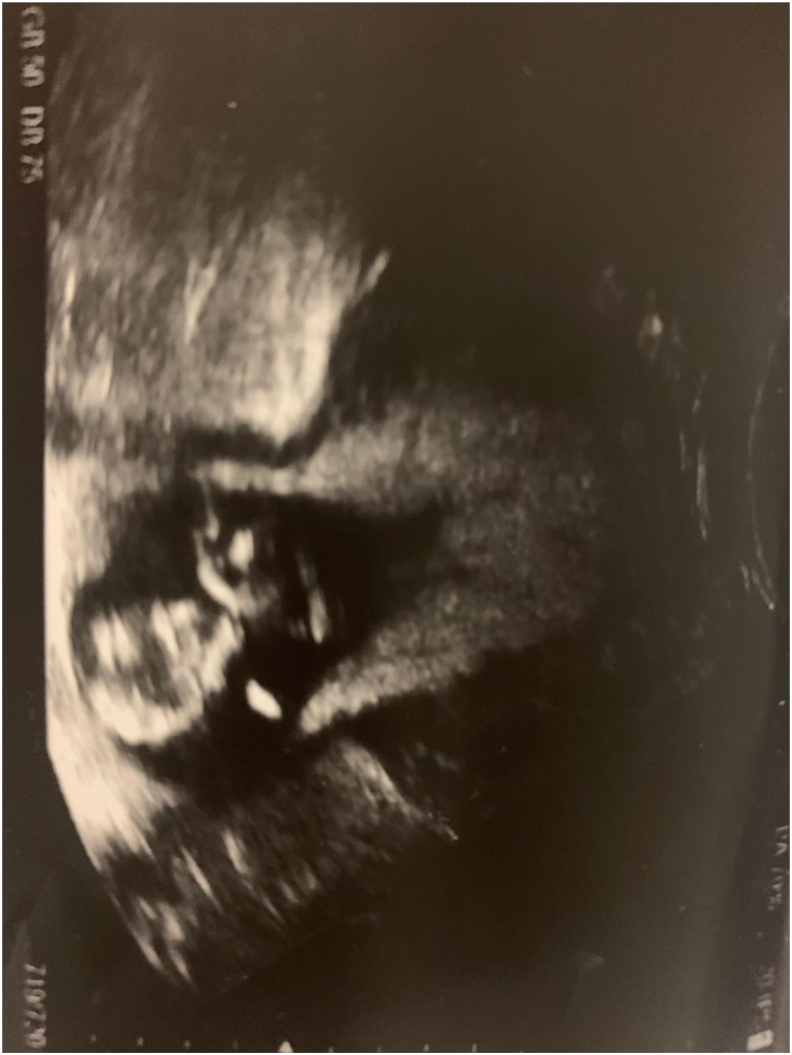
F2 abdominal ultrasound scan showing an evolving mono-fetal pregnancy with an estimated weight of 1 kg and a.

A 34 year old woman from the east of Morocco, married and mother of two children, was admitted to the emergency room with intense periumbilical and pelvic abdominal pain associated with acute vomiting without any notion of metrorrhagia. The clinical examination revealed a conscious patient with an irreducible and impulsive painful mass at the umbilical level reminiscent of a strangulated inguinal hernia (Fig. 1). We performed an abdominal ultrasound scan which showed an evolving mono-fetal pregnancy; with an estimated weight of 1 kg, and a normal amount of amniotic fluid; strangulated through an umbilical orifice (Fig. 2). Faced with this exceptional diagnosis, the case was quickly discussed in a multidisciplinary team and the decision was made to perform an MRI. It was done without injection because of the teratogenic nature of the scan (Fig. 3a, Fig. 3b).

**Fig. 3a:**
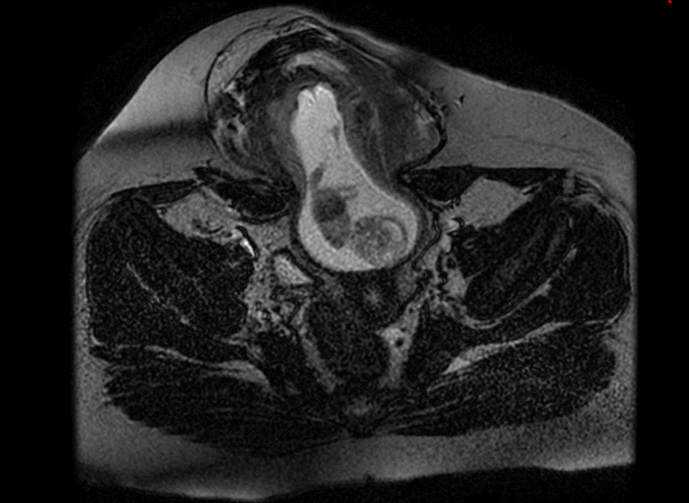
transversal cut:MRI show strangulation of a pregnant uterus through an orifice of 6 cm, resulting in a strangulated hernia of a pregnant uterus at the umbilical level, with.

**Fig. 3b:**
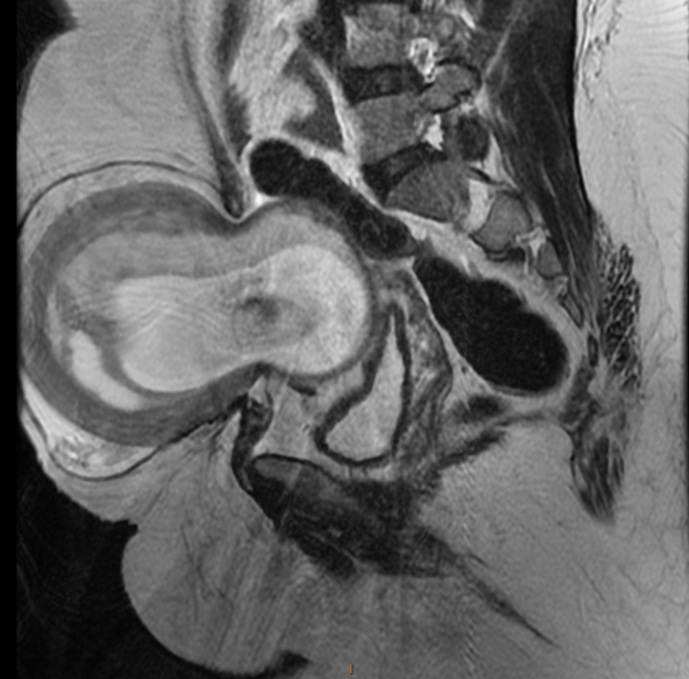
sagittal cut:MRI show strangulation of a pregnant uterus through an orifice of 6 cm, resulting in a strangulated hernia of a pregnant uterus at the umbilical level, with the.

Our radiological examination showed a strangulation of a pregnant uterus through an orifice of 6 cm, which resulted in a strangulated hernia of a pregnant uterus at the umbilical level with the right ovary. There was however no intestinal loop. After discussion with the patient, a multidisciplinary discussion was quickly made in front of this exceptional presentation and opting for a ceolioscopic exploration. It was made by the head of visceral surgery under general anesthesia was performed. It reduced the gravid uterus and the right ovary by a carefully dissection of the hernia sac and external manual assistance. Prior to the placement of the intraperitoneal plate, an obstetrical ultrasound scan had shown an evolving pregnancy (Fig. 4a, Fig. 4b, Fig. 4c).

**Fig. 4a:**
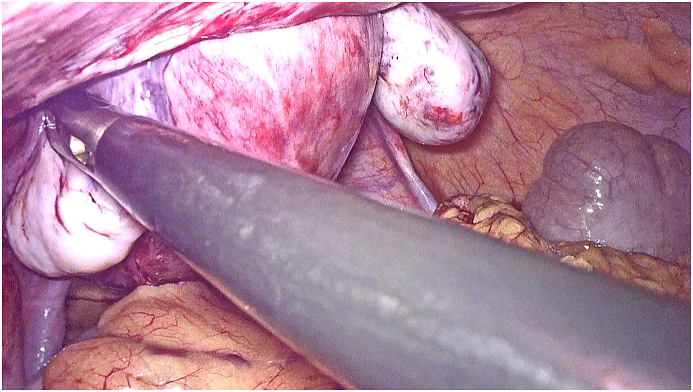
strangulated hernia of a pregnant uterus before reduction.

**Fig. 4b:**
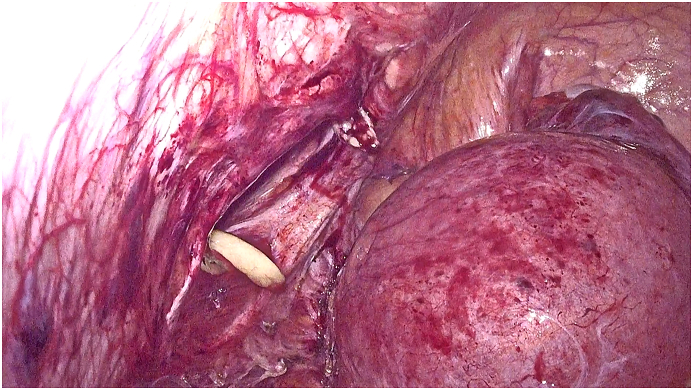
pregnant uterus after reduction and an umbilical hernia orifice measuring 6 cm.

**Fig. 4c:**
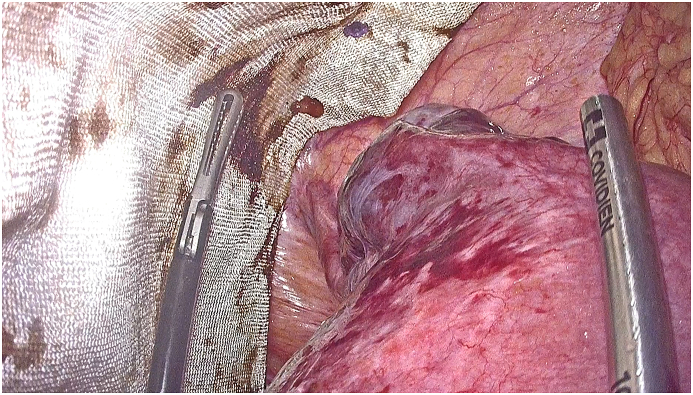
placement of the intraperitoneal plate.

The procedure went well, and it was tolerated by the patient without any adverse event allowing a good postoperative evolution The patient was discharged two days later and a cesarean section was scheduled at the end of the pregnancy, giving birth to a 2.5 kg male infant with good psychomotor development. Our patient had opted for tubal ligation, and the two-year follow-up did not show any recurrence.

## 3 Discussion

Childbirth associated with acute appendicitis presents the most solicited surgical emergencies in the world [1]. The interpretation of abdominal pain has to take into consideration first the obstetrical emergencies then other digestive and extradigestive etiologies [5].

Explained by the physiological changes and the modifications of the anatomical landmarks during pregnancy [2], as well as the non specificity of certain digestive signs [6]; a standardization of the management was imposed to protect the mother as a priority and the course of the pregnancy in second stage [7].

This maternal and fetal protection has improved recently thanks to several programs, notably the reduction in the number of pregnancies in China [8] and the adaptation of health strategies to epidemiological and economic variations [9].

The management of parietal pathology in pregnant women has been the subject of several publications in the literature which evokes causal links between pregnancy and parietal hernia according to La Place's law without having decided on the therapeutic modalities [10,11].

The same authors report an incidence of abdominal emergency of 2% of pregnancies, an incidence of umbilical hernia of 0.08% with a risk of strangulation during pregnancy of 3–5% of the patients described above [11].

We report according to SCARE guidelines [12] a case of a 34 year old patient in the first trimester of her 3rd pregnancy, followed for uncomplicated umbilical hernia and who comes for strangulated umbilical mass with intense pelvic pain.

As CT scan is the reference examination in terms of hernial strangulation [13] but considered teratogenic in pregnant women, we proceeded to perform an abdominal ultrasound showing good fetal activity and an MRI objectifying an intra hernial uterus at the umbilical level; this was based on the report of imaging during pregnancy by Flanagan E et al. [14].

Therefore, we report an exceptional case with only less than 30 reports of pregnancy strangulation reported in the literature, of which less than 10 cases of pregnancy strangulation at the umbilical level [4]. Also, given that digestive strangulation during pregnancy was rarely reported [15] and that certain obstetric differential diagnoses must be eliminated [3], we discussed the case in multidisciplinary consultation.

Consequently, we proceeded to operate the patient under general anesthesia taking into account the fetal risks [16]. Manual reduction by external approach assisted by laparoscopy was performed under visual control followed by the placement of an intraperitoneal plate to cover the umbilical orifice.

Although the current recommendations propose suture of inguinal hernias [17], we proceeded with the plate technique given the best results in terms of recurrence in pregnant women [18]. This repair of the umbilical hernia is normally required after pregnancy except in urgent cases with a risk of recurrence in the event of a subsequent pregnancy [19]. Some authors describe a risk of recurrence in women of childbearing age of 12%, however they do not propose systematic surgical treatment [20]; while others propose treating the umbilical hernia at the same time as the caesarean section without any repercussions on maternal and infant morbidity [19].

## 4 Conclusion

In fact, our main goal through this work is to report the parietal physiological changes during pregnancy, and to focus on hernial strangulation, especially uterine strangulation as an exceptional incident complicating pregnancy.

## Patient perceptive

The procedure of surgery was explained to the patient with all advantages and possible complications. He agreed on the procedure and informed consent was taken from her.

## Sources of funding

The author(s) received no financial support for the research, authorship and/or publication of this article.

## Ethics approval

Not applicable.

## Consent of patient

Written informed consent was obtained from the patient for publication of this case report and accompanying images. A copy of the written consent is available for review by the Editor-in-Chief of this journal on request.

## Author's contribution

Jabi Rachid: Writing, review and editing of the manuscript. Siham Elmir, Karam Saoud, Houda Mirali, Siham Nasri: Contributed for diagnose and treatment of the patient. Mohamed Bouziane, Skiker Imane, Hanane Saadi, Brahim Housni: Review, Supervision and surgeons of the patient.

## Trail registry number

Our paper is a case report; no registration was done for it.

## Guarantor

Jabi Rachid.

## Provenance and peer review

Not commissioned, externally peer-reviewed.

## Declaration of competing interest

The authors declared no potential conflicts of interests with respect to research, authorship and/or publication of the article.
